# Satellite data track spatial and temporal declines in European beech forest canopy characteristics associated with intense drought events in the Rhön Biosphere Reserve, central Germany

**DOI:** 10.1111/plb.13391

**Published:** 2022-01-27

**Authors:** E. West, P. J. Morley, A. S. Jump, D. N. M. Donoghue

**Affiliations:** ^1^ Department of Geography Durham University Durham UK; ^2^ Faculty of Natural Sciences Biological and Environmental Sciences Stirling University Stirling UK

**Keywords:** Anomaly, drought, forest health, NDVI, remote sensing

## Abstract

- The increasing intensity and frequency of droughts under climate change demands effective ways to monitor drought impacts. We sought to determine how different satellite remote sensing sources influence our ability to identify temporal and spatial impacts on European beech forest canopy health during intense drought events.
- Imagery from three satellite series (MODIS, Landsat and Sentinel‐2) was used to observe changes in canopy health during the intense droughts of 2003 and 2018 in the Rhön Biosphere Reserve, central Germany. Monthly normalized difference vegetation index (NDVI) anomalies were calculated for each satellite between 2000–2020 and compared against temperature, precipitation and the standardized precipitation evapotranspiration index (SPEI).
- Severe canopy impacts in 2003 and 2018 were associated with low NDVI in August and September. At the stand‐scale, Sentinel‐2 data allowed a spatially detailed understanding of canopy‐level impacts, while MODIS provided the clearest temporal progression of the drought’s impacts on the forest canopy. Low NDVI values were not exclusively associated with extremes of either temperature and precipitation individually; however, low canopy NDVI in August was associated with SPEI values below −1.5.
- Although the intense drought of 2018, as defined by meteorological parameters, peaked in July, canopy NDVI did not decline until August, highlighting that our ability to detect canopy impact during drought events is sensitive to the timing of image acquisition. No single satellite sensor affords a full picture of the temporal or spatial progression of drought impacts. Consequently, using sensors in tandem provides the best possible representation of canopy health during intense drought events.

## Graphical abstract

Satellite NDVI data is able to capture drought induced canopy stress in European beech forests, but is sensitive to the timing of expression and a combination of data sources is needed to understand both temporal and spatial patterns of canopy stress.

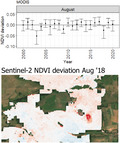

## INTRODUCTION

Globally, droughts are becoming more intense and frequent (Dai 2011; Erfurt *et al*. 2019). Future climate projections predict that extremely hot and dry seasons will increase in frequency towards the end of the century (Knapp *et al*. 2015; IPCC 2018; Konapala *et al*. 2020). Central Europe has seen several major drought events thus far in the 21^st^ century (Bréda *et al*. 2006; Boergens *et al*. 2020; Ionita *et al*. 2021). Analysis of drought indices reveals that recent European droughts are not unusual when placed in the context of precipitation records for the last 170 years (Vicente‐Serrano *et al*. 2021). However, indices based on precipitation alone do not effectively quantify the impact that precipitation deficits have when they coincide with elevated temperatures. Drought indices that account for both precipitation and temperature find that there has been a greater frequency of intense droughts from 2000 onwards because of hotter background temperatures (Erfurt *et al*. 2019). The intense drought event of 2003 is often cited as the benchmark for ‘hotter’ drought in Europe, with precipitation deficits of −200 mm and a mean growing season temperature of 2.1 °C above the average (Schuldt *et al*. 2020). In 2018 the Central European mean growing season temperature was 3.3 °C above the average, which coincided with a climatic water balance anomaly of −238 mm, resulting in the most intense and long‐lasting summer drought ever recorded in this region (Schuldt *et al*. 2020).

Intense drought events, such as those in 2003 and 2018, impact forests in a number of ways, ranging from leaf wilting and early senescence to tree mortality (Jump *et al*. 2017). While temperate forest ecosystems are adapted to withstand occasional heat stress and water deficits, the combination of high temperatures and lack of precipitation during the growing season is extremely damaging to leaf function (Zhang *et al*. 2004; Zaitchik *et al*. 2006; Vicente‐Serrano *et al*. 2013; Allen *et al*. 2015). The disruption to photochemical reactions within the leaves leads to premature leaf discolouration and shedding in response to hot drought events (Wang *et al*. 2018). In addition to phytochemical responses, the increased transpiratory demand that coincides with hot drought events can also trigger stomatal closure, further limiting carbon uptake (Adams *et al*. 2009; Liu *et al*. 2019). Consequently, intense drought events cause a reduction in gross primary production during the drought year, which can have long‐term impacts on forest growth rates, reducing ecosystem productivity and the ability of forests to store and sequester carbon (Ciais *et al*. 2005). Reports of intense drought events acting as a trigger for tree mortality are also increasingly common (Allen *et al*. 2010; Greenwood *et al*. 2017; Senf *et al*. 2020). While the risk of drought‐induced growth decline and mortality was thought to be most severe at the hotter and drier margins of a species distribution, recent drought‐induced die‐off events have demonstrated unexpectedly high vulnerability to growth decline in the core areas of species distributions (Allen *et al*. 2015; Cavin & Jump 2017; Vilà‐Cabrera & Jump 2019).

The earliest visible signs of drought‐induced forest stress are leaf discolouration and early senescence at the canopy level. However, drought‐induced canopy decline can be spatially variable, with differing degrees of impacts both within tree stands as well as at larger forest or continental scales. Consequently, identifying the spatial and temporal variation in drought‐induced forest impacts is vital to improve our understanding of drought‐driven growth decline and mortality, and the factors that increase forest vulnerability to drought. Satellite imagery is useful for monitoring drought impacts on forest canopies due to its consistent repeat measurements over large areas that enable non‐invasive examination of tree canopies at continental scales (Verbesselt *et al*. 2010). The Normalized Difference Vegetation Index (NDVI) is the most widely used vegetation index in studies linking forest canopy condition to drought events, with declines in NDVI occurring during drought years in response to changes in precipitation and temperature (Recanatesi *et al*. 2018; Philipp *et al*. 2021). NDVI is ubiqitous amongst studies using remote sensing data to characterize environmental change because it can be calculated from multiple different sensors, including imagery from the freely available MODIS, Landsat and Sentinel‐2 archives. However, the spatial and temporal characteristics of each sensor differ, which may impact our interpretation of NDVI change and bring different advantages for detecting drought‐induced canopy impacts.

The MODIS imagery has been the most widely used remote sensing imagery for studying the impacts of drought on forest canopy condition. MODIS has the best temporal resolution of any freely‐available satellite dataset, with a 1–2 day revisit time and an archive spanning back to 2000, enabling canopy impacts to be quantified covering every major drought event in the 21^st^ century. However, the spatial resolution of optical bands from the MODIS sensors used for common vegetation indices can have a pixel size up to 500‐m wide, making it difficult to account for heterogeneity in the drought response at finer spatial scales (Zaitchik *et al*. 2006; Buras *et al*. 2020). In contrast, Landsat’s spectral bands in the visible and shortwave infrared regions have recorded reflectance at a pixel size of 30 m since 1982, enabling a longer time record for comparing drought impacts against historical conditions. However, the satellites in the Landsat family have a longer revisit time of 16 days, thereby reducing the number of images available to examine changes in canopy condition that can be short‐lived (Zhu 2017; Nguyen *et al*. 2020). Sentinel‐2 further improves upon the spatial resolution of the Landsat family of sensors with its 10 m pixel size. In addition, the Sentinel‐2 constellation of two satellites gives a 5‐day revisit time, thereby increasing the number of images available, but lacks an extensive legacy, having only been launched in 2015, making it difficult to compare drought events to baseline conditions. While the potential benefit of higher spatial resolution satellite imagery has been recognized, there are very few direct comparisions between sensors with different spatial and temporal characteristics (Buras *et al*. 2020). Therefore, there is a lack of clarity on how the different attributes of these sensors will impact our ability to identify temporal and spatial impacts of intense drought events on forest canopies, hindering the development of early‐warning indicators of forest dieback (Camarero 2021).

European beech *(Fagus sylvatica)* is a dominant broadleaf tree species in Central Europe that has broad importance across commercial forestry, biodiversity and cultural values. European beech is vulnerable to drought because it maintains a larger leaf area than most hardwood species and has shallow root systems which restrict its access to deep soil moisture (Leuschner 2020). Following the 2003 and 2018 drought events, European beech trees showed premature leaf discolouration and shedding, as well as widespread, but highly heterogeneous mortality across Central Europe (Bréda *et al*. 2006; Schuldt *et al*. 2020). The high vulnerability of European beech to drought in combination with future climate projections for increasingly frequent and intense drought events highlights the need to develop monitoring protocols for beech‐dominated forests. Consequently, in this research we aimed to address the following key questions: (i) how does NDVI response develop over time during an intense drought event; (ii) how does the canopy level NDVI response relate to the timing of intense drought events; and (iii) what influence does the spatial and temporal resolution of satellite sensors have on the ability to detect a drought signal in the forest canopy?

## MATERIAL AND METHODS

### Study area

In April 2018, Central Europe saw the beginning of a high‐pressure system which persisted until October 2018 (Buras *et al*. 2020). This weather system resulted in a drought which was both long‐lasting and the most intense on record in this region, surpassing the previous record set during the 2003 European drought (Buras *et al*. 2020). The 2018 drought had the largest water deficit in Central Europe in the 21^st^ century and coincided with unusually high temperatures, resulting in an intense event characteristic of the ‘hotter’ droughts anticipated under ongoing climate change (Allen *et al*. 2010; Buras *et al*. 2020; Senf *et al*. 2020). While the 2018 drought in isolation was extreme, a second drought in 2019 compounded its impacts, thus retarding vegetation recovery (Boergens *et al*. 2020; Schuldt *et al*. 2020).

**Fig. 1:**
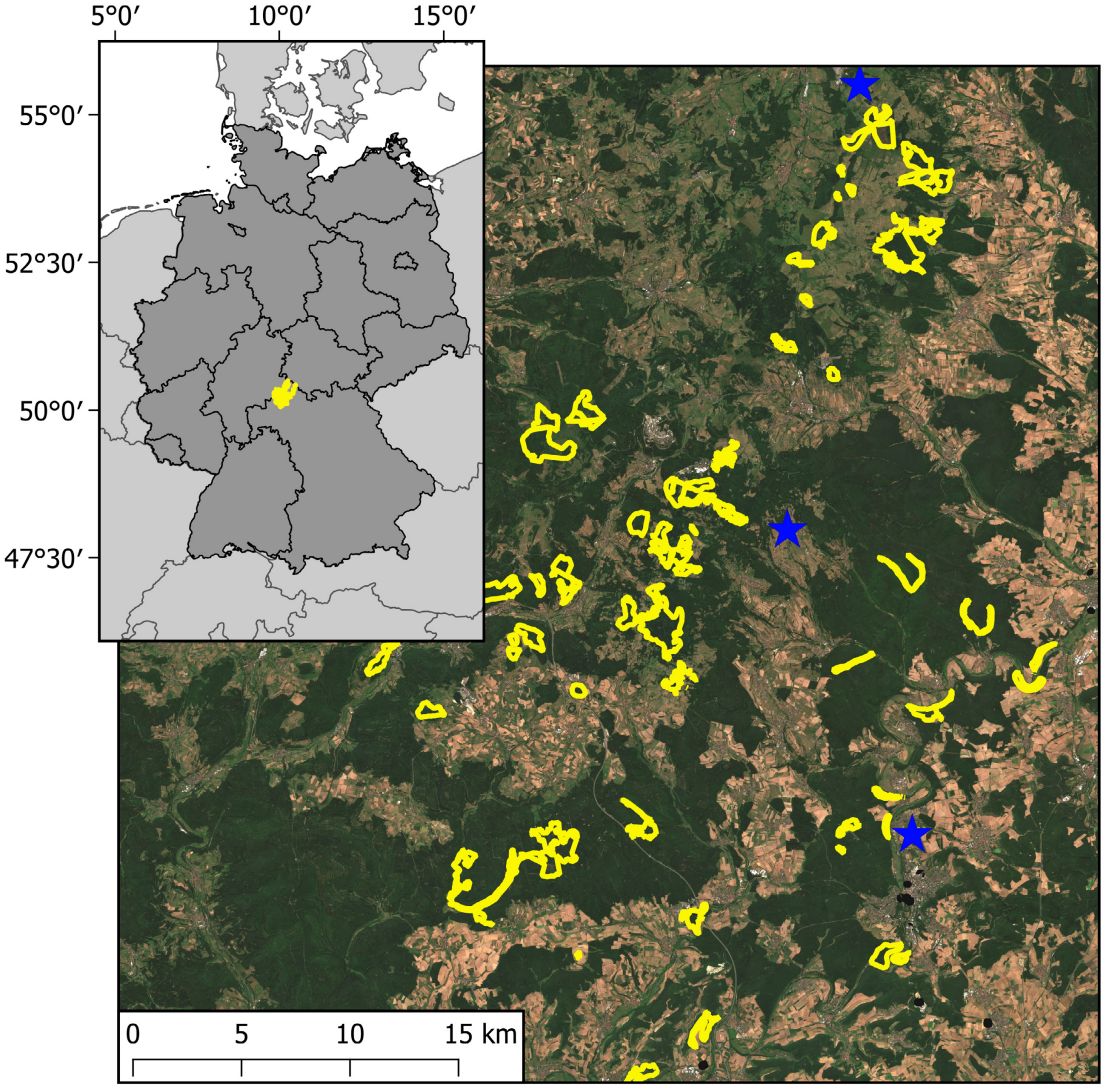
Location of the study area in the Rhӧn Biosphere Reserve in central Germany (inset), imagery from Sentinel‐2 during the summer of 2018. Yellow boundaries show the core areas of the Rhӧn Biosphere Reserve in Bavaria and stars are locations of the climate stations.

The intense droughts of 2003, 2018 and 2019 severely impacted European beech forests in Central Europe, resulting in widespread defoliation and a reduction in leaf chlorophyll content. Severe and unexpected impacts were observed in core regions of the species’ distribution, including in northern Bavaria, Germany, where this species is predicted to show stable growth and be suited to future climatic conditions (Felbermeier 1994; Kӧlling 2007). In the Rhӧn Biosphere Reserve (Fig. 1), tree mortality following the 2018/2019 drought events was widespread, yet highly heterogeneous, resulting in a gradient in the severity of drought impacts (Obladen *et al*. 2021). The UNESCO Rhӧn Biosphere Reserve is located in Central Germany and spans three states: Bavaria, Hesse and Thuringia (Fig. 1). The Biosphere Reserve covers 72,802 ha of northern Bavaria, with approximately 2.3% of the reserve designated as core areas that do not receive any intervention management and are home to several species of endangered flora and fauna (UNESCO 2021). The low mountainous forests within the Rhӧn Biosphere Reserve are dominated by European beech, with twice the national average of beech occurrence, and are not subject to intervention management. Consequently, impacted trees were not removed following the drought events, enabling forest recovery and sustained canopy impacts to be assessed; a task which would not be possible in the surrounding production forests where impacted stands were felled to avoid economic losses due to the commercial value of the timber.

### Climate data

Daily precipitation and temperature measurements spanning 2000–2020 from three weather stations, Birx/Rhӧn, Sandberg and Bad Kissingen (Fig. 1), were downloaded from the German Weather Service (Deutscher Wetterdienst 2020). The weather stations are approximately 35 km apart and at altitudes between 745 m and 280 m a. s. l. Daily mean and maximum temperatures were averaged to calculate monthly mean and maximum temperatures between 2000 and 2020. Daily precipitation data were summed to return the monthly total precipitation between 2000 and 2020.

#### Standardized Precipitation Evapotranspiration Index (SPEI)

The SPEI is a drought indicator used to determine deviations from average water balance over a specified time frame (Vicente‐Serrano *et al*. 2013). Vegetation has been found to be predominantly responsive to short‐term drought time scales, hence a 3‐month integration period was determined as an appropriate time scale for contextualizing vegetation health in response to seasonal drought (Vicente‐Serrano *et al*. 2013, 2020). Daily temperature minima and maxima were averaged to calculate monthly potential evapotranspiration (Buras *et al*. 2020). The Hargreaves equation (Hargreaves 1994) was found to strike a useful balance between minimal data requirements and sensitivity (Vangelis *et al*. 2013; Stagge *et al*. 2014). SPEI was calculated using a 3‐month integration period in R using the *SPEI* package (Vicente‐Serrano *et al*. 2010; Beguería *et al*. 2014).

### Satellite data

#### Moderate Resolution Imaging Spectroradiometer (MODIS)

The MODIS sensor captures data in 36 spectral bands at spatial resolutions (pixel size) of 250 m (2 bands), 500 m (5 bands) and 1 km (29 bands). In combination, the two satellites (Terra and Aqua) acquire repeat imagery across the globe every 1–2 days, providing large‐scale observational data (Table 1). Due to the frequency of cloud cover within optical satellite imagery, MODIS products are provided in composites that take the average surface reflectance from a regular 8‐day window, masking cloud and cloud shadow and retaining the best available data. The MOD09Q1 version 6 250‐m surface reflectance data in the red and near‐infrared bands were used here, with further data quality control implemented to only retain pixels that are labelled as ‘cloud free’ and ‘ideal quality’ across all bands in the MODIS quality assurance bands provided with 8‐day composites. A linear model of NDVI against time was used to assess the presence or absence of a greening trend in the MODIS time series. Here we found no significant trend in the NDVI time series, and so the absolute values of NDVI from MODIS were used in subsequent analyses.

**Table 1:** Sensor temporal and spatial characteristics from the satellite datasets used in this study. The pixel sizes reported are for the visible and near‐infrared spectral bands that were used to calculate NDVI.

	Satellite sensor				
	MODIS	Landsat TM	Landsat ETM+	Landsat OLI	Sentinel‐2 MSI
Revisit time	1–2 days	16 days	16 days	16 days	2–10 days
Mission dates	1999–	1984–2012	1999–	2013–	2015–
Pixel size	250 m	30 m	30 m	30 m	10 m
Red band width (nm)	620–670	630–690	630–690	640–670	650–680
NIR band width (nm)	841–876	760–900	770–900	850–880	790–880

#### Landsat

Since the launch of Landsat 4 in 1982 and Landsat 5 (TM) in 1984, the Landsat series of satellite sensors have collected data at 30‐m pixel size. With the launch of Landsat 7 (ETM+) in 1999 and Landsat 8 (OLI) in 2013, the Landsat archive provides the longest continuous record of optical satellite imagery, with a revisit period of 16 days (Table 1). In this study, all available Tier 1 surface reflectance images from a common period with MODIS (2000–2020) were used to calculate monthly data composites. Landsat data were processed to surface reflectance using the Landsat Ecosystem Disturbance Adaptive Processing System (LEDAPS) for Landsat TM and ETM+ and the Land Surface Reflectance Code (LaSRC) for Landsat OLI, with clouds and cloud shadows masked from the resultant surface reflectance data using the pixel quality assurance information provided with surface reflectance data in the Google Earth Engine image collections. A linear model of NDVI against time highlighted a significant positive greening trend for the forest areas of the Rhӧn Biosphere Reserve in the Landsat NDVI record that was not observed in either the MODIS or Sentinel‐2 NDVI response (Figures S1–S3). While there is no clear pattern in the Landsat time series that would explain the presence of the greening trend yet the absence of the trend in the MODIS and Sentinel‐2 records, to avoid a mis‐estimation of anomalies, the Landsat time‐series data were first detrended on a per‐pixel basis by fitting a linear model of NDVI against time and subtracting the fitted trend from the observed NDVI values. Detrending is carried out using the full annual response of cloud‐free pixel values with anomaly calculations then applied to this detrended series.

#### Sentinel‐2

The Copernicus Sentinel‐2 MSI sensor collects optical data at 10 m (4 bands in visible and near‐infrared regions) and 20 m (6 bands in the red‐edge and shortwave infrared regions) spatial resolution from a pair of satellites with a combined 5‐day revisit period. The first of these, Sentinel‐2A, was launched in 2015, with Sentinel‐2B following in 2017. Since the launch of Sentinel‐2B, processed data have been available at surface reflectance using the sen2cor processor. Here we use all the pre‐processed surface reflectance data available since the launch of Sentinel‐2B in March 2017. Consequently, the Sentinel‐2 archive covers a very short time period but affords a higher spatial resolution than the other sensors and a shorter temporal revisit period than Landsat (Table 1). Cloud and cloud shadow are masked from Sentinel‐2 images using the Sentinel‐2 Cloud Probability image collection available in Google Earth Engine. Sentinel‐2 Cloud Probability layer is produced by Sentinel Hub and provides a probabilty of each 10‐m pixel being cloudy with pixels with high values likely to contain clouds. A threshold of 10% chance of being a cloudy pixel is used in this study because this enables the removal of cirrus clouds, which distort the NDVI response and would return a false estimate of canopy impact. The position of cloud shadows is estimated using the solar azimuth angle for each image to project the position of the clouds onto the surface of the Earth. A linear model of Sentinel‐2 NDVI against time found no significant greening trend in the NDVI time series, so the absolute values of NDVI from Sentinel‐2 were used in subsequent calculation of anomalies.

All the surface reflectance data across the three satellite series used in this study were processed in Google Earth Engine (Gorelick *et al*. 2017), with cloud and cloud shadow removed. Every available image (or 8‐day composite in the case of MODIS) was used to calculate the monthly mean NDVI reponse per pixel. Monthly NDVI data were subsequently masked to exclude areas outside of the the core areas of the Biosphere Reserve, where management of the forest differs. This boundary layer provided a high level of precision in marking the forest boundary but does not make a distiction between forest types. Therefore, the 2018 Copernicus CORINE Land Cover map (Copernicus 2017) was used to to retain only areas classified as broadleaf forest. The CORINE land cover map provides information for Europe’s land cover at a pixel size of 100 m, specifing 44 classes of land cover at an accuracy of over 85% (European Environment Agency 2019). The combination of protected area boundaries and the CORINE land cover map ensures that the spectral response of managed broadleaf forests, conifer forests, grasslands and arable farmland did not influence the landscape‐scale spectral response, and thus emphasized the spectral response of the European beech forest. Across all of the core areas of the Biosphere Reserve this mask results in a maximum number of possible cloud‐free broadleaf forest pixels of 499 for MODIS, 34,911 for Landsat and 313,009 for Sentinel‐2 per image.

#### Anomalies in NDVI

**Fig. 2:**
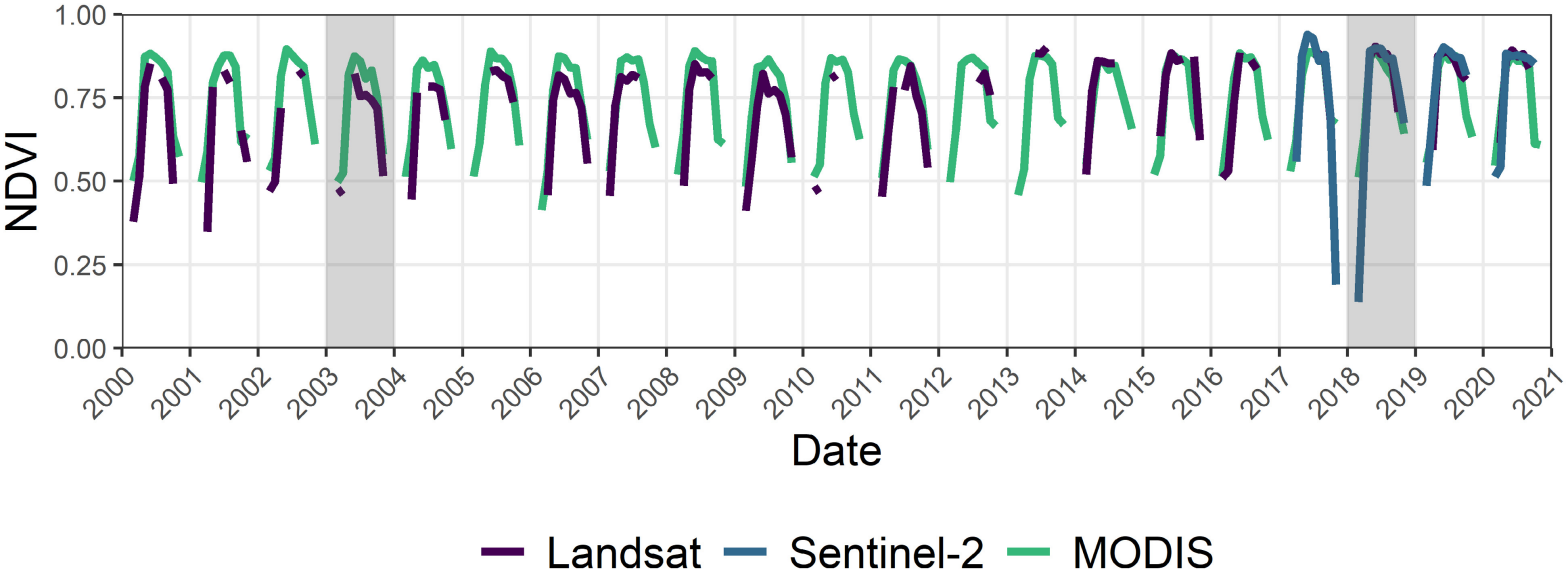
Time series NDVI data from MODIS, Landsat and Sentinel‐2 satellite series between 2000 and 2020. The intense drought years of 2003 and 2018 are shaded in grey.

Here we assess the temporal and spatial response of forest canopy to the intense drought events of 2003 and 2018 using the NDVI. While the NDVI response between all three sensors studied here is similar (Fig. 2), slight differences in the position and width of the red and near‐infrared spectral bands between sensors (Table 1) means that we cannot make direct comparisons of NDVI values from the different sensors. As such, the focus of this paper is to identify how the sensor properties (revist time and pixel size) impact our ability to track drought impacts on European beech forests and identify spatial variation in the severity of impacts across a landscape. To identify the temporal response, a baseline NDVI value of the forest canopy for each month in the growing season is defined as the median value of all the cloud‐free broadleaf forest pixels across the available time series. Monthly anomalies are then calculated for each year to identify how median canopy response deviates from the long‐term baseline. To assess the association between climate and the temporal response of the canopy, monthly mean and maximum temperature, summed precipitation and the SPEI were compared against the monthly NDVI values from the MODIS time series. To identify heterogeneity in the stand‐level response of the forest canopy, deviation from the monthly baseline was calculated per pixel to return the spatial pattern of canopy anomalies.

## RESULTS

**Fig. 3:**
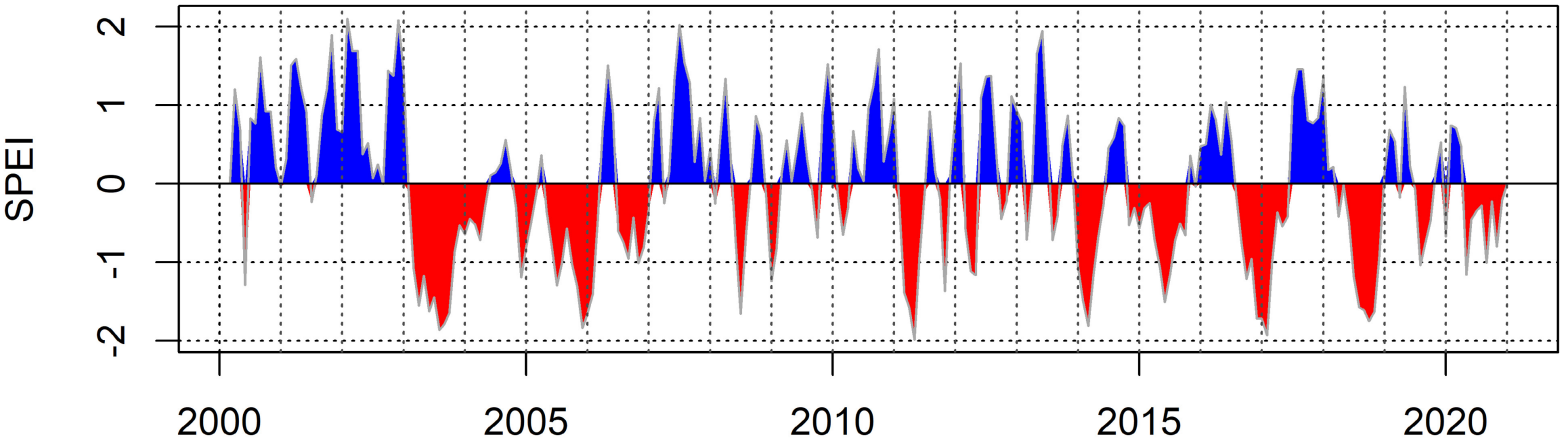
Standardized Precipitation Evapotranspiration Index (SPEI) between 2000 and 2020 from northern Bavaria, Germany. SPEI was calculated using a 3‐month time scale with negative (red) values indicating drier conditions due to a higher evaporative potential, typical of summer conditions, while positive values (blue) indicate lower evaporative potential, as is common during winter. The threshold used to indicate a drought is −1 and an intense drought is −2 (Potop *et al*. 2014).

During the 2018 drought, the SPEI value at the Rhӧn Biosphere Reserve first falls below −1 in July and remains below −1.5 until November. During the subsequent 2019 drought, SPEI remains below 0 for the majority of the year, falling below −1 in July, whilst being visibly less severe than that of 2018 (Fig. 2). Similarly, in 2003 the SPEI reached −1.5 in June and remains below −1 until the following year. The 2003 event was visibly very long‐lasting, with an earlier onset of low values than its 2018 counterpart. Although neither event reached values as low as −2, with the absolute lowest values seen in 2018 similar to those of 2003, the 2003 event was longer‐lasting. Whilst spikes of very low SPEI are present in other years, notably 2005/06, 2011 and 2016/17, these events are short‐lived and occur outside of the growing season (Fig. 3). Therefore, these events do not combine the severe intensity as well as the long duration of drought conditions during the growing season which made the 2003 and 2018 droughts so damaging.

### The NDVI anomalies

**Fig. 4:**
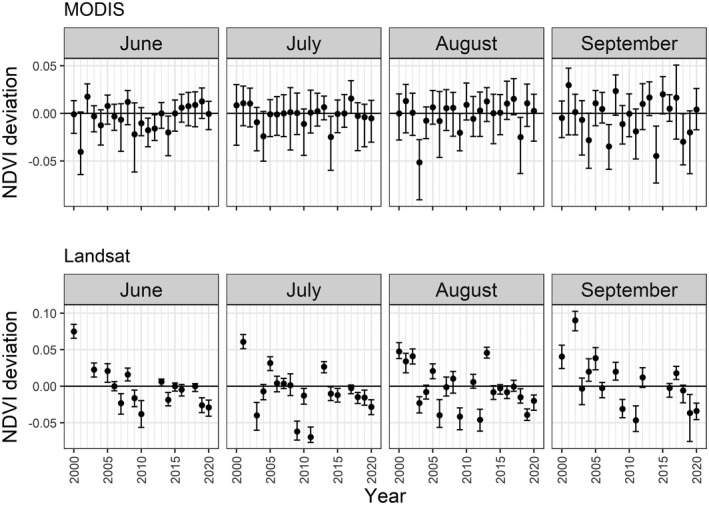
Annual NDVI deviation (median and interquartile ranges) for the European beech forests in the core protected areas of the Rhӧn Biosphere Reserve from MODIS 8‐day composites (top row) and detrended Landsat image (bottom row) between May and September. Positive deviations indicate annual median values are above the long‐term average derived from all cloud‐free broadleaf forest NDVI values, while negative deviations indicate annual NDVI values below average indicating reduced canopy vigour.

**Fig. 5:**
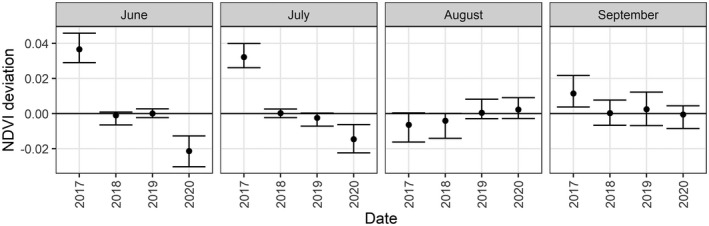
Annual NDVI deviation (median and interquartile ranges) for the European beech forests in the Rhӧn Biosphere Reserve from Sentinel‐2 images between June and September. Positive deviations indicate annual median values are above the long‐term average derived from all cloud‐free broadleaf forest NDVI values, while negative deviations indicate annual NDVI values below average indicating reduced canopy vigour.

The NDVI anomalies from the MODIS time series show that canopy response during the 2018 drought first drops below average in July but declines severely at the landscape‐scale in August, continuing into September (Fig. 4). A considerable decline in MODIS NDVI was also seen during the 2003 European drought in August, returning to average conditions by September 2003. A similar pattern is observed in the Sentinel‐2 time series, with the strongest decline in average landscape‐scale NDVI during August 2018 (Fig. 5). However, the Landsat time series does not identify strong declines in landscape‐scale NDVI during the 2018 drought event, instead estimating the average landscape value close to the long‐term baseline and showing greater inter‐annual variability than MODIS (Fig. 4).

**Fig. 6:**
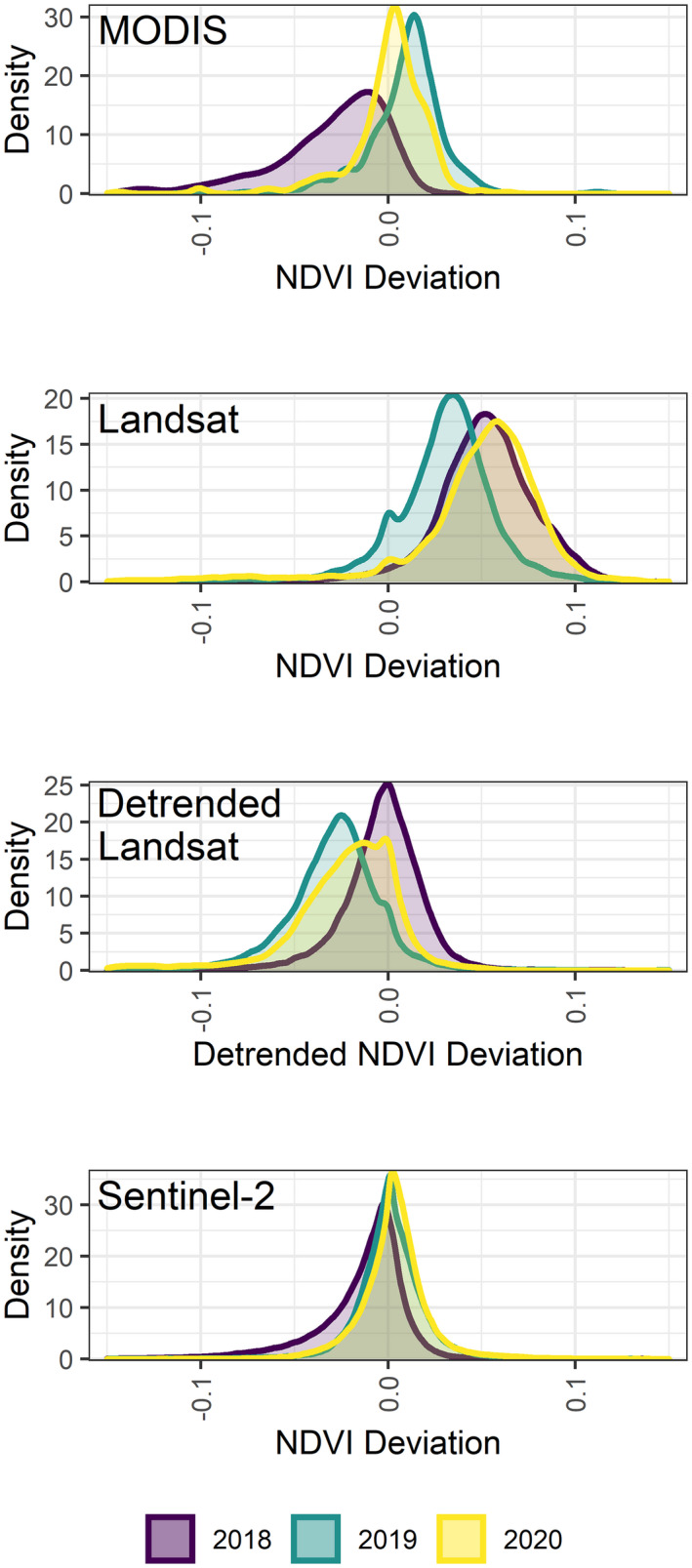
Frequency distribution of NDVI deviation values during August for the broadleaf forests within the core protection areas of the Rhӧn Biosphere Reserve in northern Bavaria, Germany. NDVI deviation is measured as the difference between each year and the median of all cloud‐free broadleaf forest NDVI pixel values recorded between 2000–2020 for the MODIS and Landsat series, and from 2017–2020 for Sentinel‐2. Distribution curves are shown for the August NDVI values during the 2018 and 2019 drought events and subsequent recovery in 2020.

The canopy response of European beech forests to intense drought is most obvious in August during the drought year. Whilst the average landscape‐scale deviation is often small (the median deviation of MODIS NDVI in August 2018 is −0.057 compared to the baseline of 0.864), there is a substantial shift in the distribution of NDVI values resulting in a long tail of negative deviation values present in August 2018 (Fig. 6). This shift is replicated by MODIS and Sentinel‐2, with a similar but less intense response during 2019 and a return to the baseline distribution of NDVI values in 2020 (Fig. 6). However, the distribution response of NDVI from Landsat does not match those of Sentinel‐2 or MODIS, which instead suggest close to average conditions in 2018, followed by declining canopy condition in 2019 (Fig. 6).

**Fig. 7:**
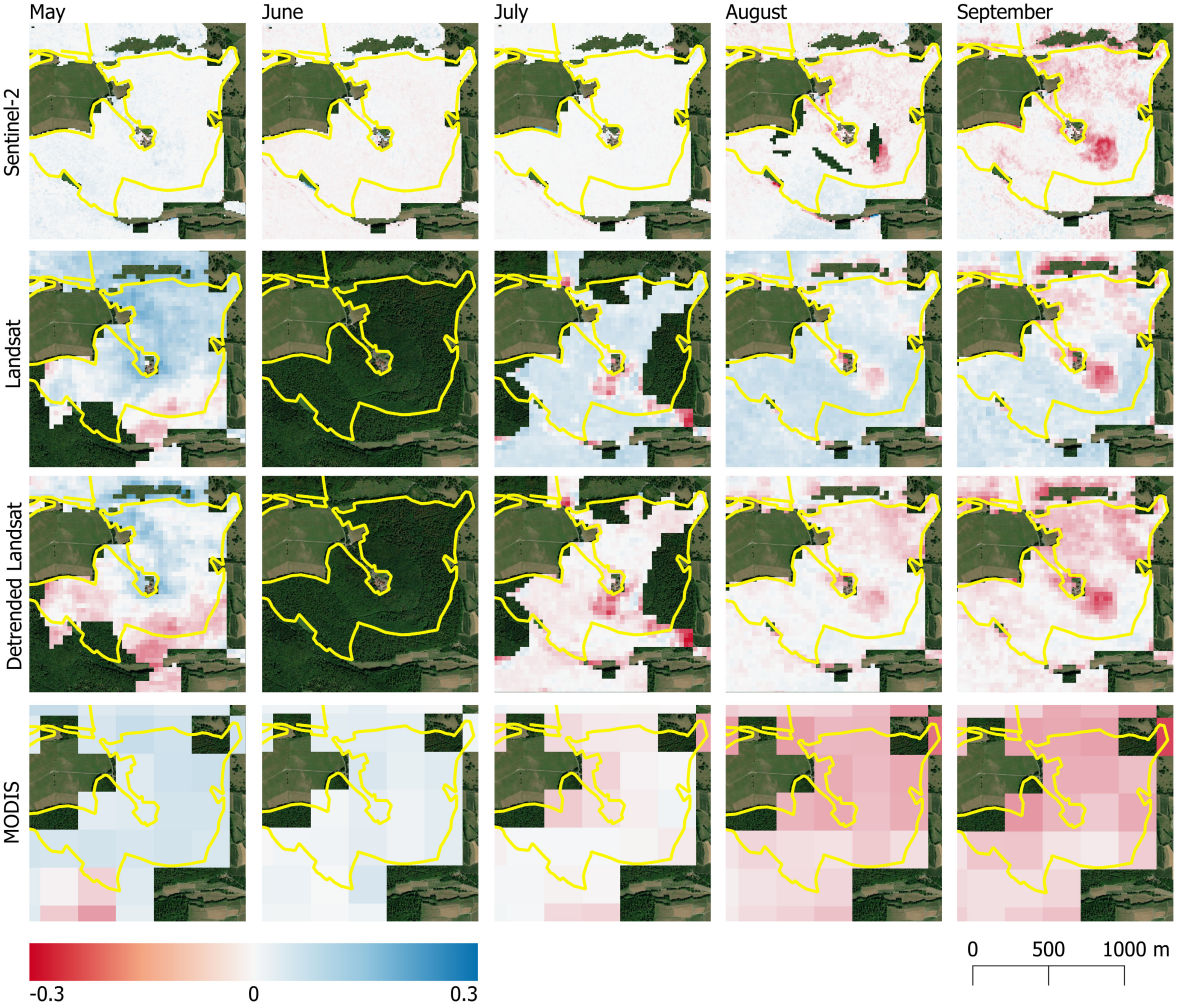
Spatial pattern of NDVI deviations between May–September 2018 of the broadleaf forest in a subset of the the Rhӧn Biosphere Reserve from MODIS, Landsat, detrended Landsat and Sentinel‐2. Monthly NDVI anomalies are deviations from the monthly baseline, which is determined as the median NDVI value from European beech forests in the Biosphere Reserve between 2000 and 2020 (2017–2020 for Sentinel‐2). Yellow boundaries show the core areas of the Rhӧn Biosphere Reserve in Bavaria.

While MODIS returns the best temporal estimate of intense drought impacts on the forest canopy, the relatively coarse spatial resolution means that spatial patterns of canopy decline are poorly represented (Fig. 7). Both the detrended Landsat NDVI and Sentinel‐2 NDVI anomalies return a more spatially precise estimate of impacts and reveal local hotspots of canopy decline. Monthly NDVI deviations show the forest canopy responding strongly to the 2018 drought event in August, with low NDVI values continuing through September (Fig. 7). The spatial patterns revealed by Landsat and Sentinel‐2 show that the forest canopy is not uniformly impacted by the intense drought event of 2018. Instead, the forest displays a patchy response, with hotspots of decline in canopy NDVI amongst a broader, but less severe, background decline (Fig. 7).

**Fig. 8:**
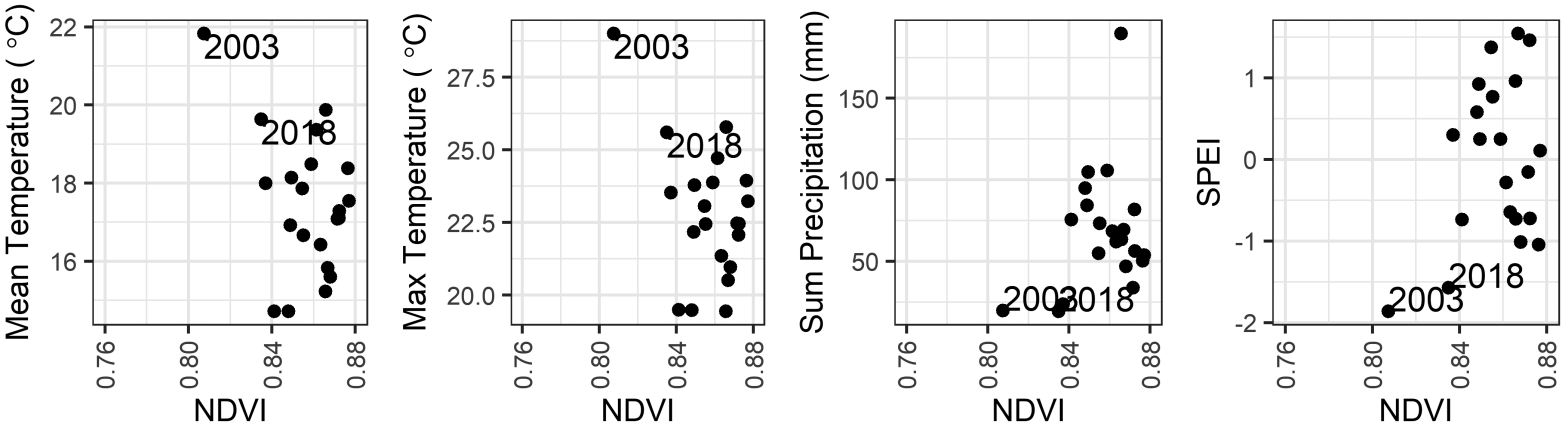
Association between climate variables on MODIS median August NDVI from European beech forests in the Rhӧn Biosphere Reserve. The averaged daily mean and maximum temperatures and summed precipitation were calculated for August and the Standardized Precipitation Evapotranspiration Index was calculated over a 3‐month window up to August.

**Fig. 9:**
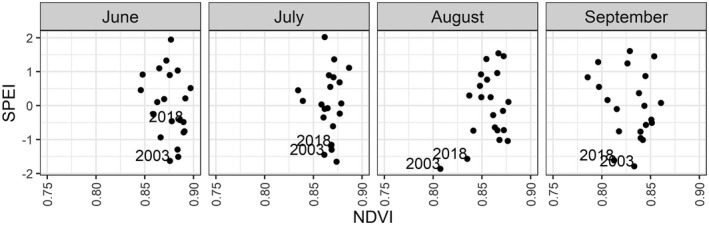
Association between Standardized Precipitation Evapotranspiration Index (SPEI) and MODIS median monthly NDVI (June–September) from European beech forests in the Rhӧn Biosphere Reserve. SPEI was calculated using a 3‐month period.

Comparisons of climate variables against the median monthly MODIS NDVI values show that reduced NDVI does not uniformly coincide with extreme high temperatures nor with low precipitation. By contrast, particularly low SPEI values (< −1.5) coincide with low NDVI values (Fig. 8). While SPEI values indicate that the drought events of 2018 and 2003 began in July 2018 and June 2003, with SPEI values falling below −1 in these months, the average NDVI response of the beech forest does not decline until August for both years, indicating a delay between the onset of the drought and the expression of canopy‐level impacts detectable in satellite remote sensing data (Fig. 9).

## DISCUSSION

Our understanding of the spatial and temporal variation of drought‐associated forest canopy impacts varies, depending on the satellite data used to derive NDVI anomalies. Temporal patterns in NDVI decline associated with the 2003 and 2018 droughts are clear when observing the MODIS NDVI anomalies, which show that declines in the canopy NDVI began in July, became clearest in August, and persisted until September during both drought years within the Rhӧn Biosphere Reserve (Fig. 4). While MODIS gives the best temporal indicator that European beech forests are under stress from intense drought events, the coarse pixel size of the MODIS data is insufficient to provide a picture of spatial heterogeneity in the canopy impacts (Fig. 7). In contrast, Landsat and Sentinel‐2 NDVI anomalies provide a much more detailed spatial representation of canopy‐level impacts of European beech in response to intense drought (Fig. 7). Consequently, a combination of data from different sources is required to resolve both temporal and spatial patterns of drought impacts on forest canopies.

The distribution of MODIS and Sentinel‐2 data demonstrate long tails of negative NDVI anomaly values in August, corresponding to reduced canopy health (Fig. 6). However, this distribution is not replicated with Landsat. Despite proving useful for other forestry applications, care needs to be taken when using Landsat imagery for observing fine phenological details of forest canopies, because Landsat’s 16‐day revisit time hinders its collection of cloud‐free imagery (Hilker *et al*. 2012; Hamunyela *et al*. 2016; Kowe *et al*. 2020). Across the 20‐year study period, 33% of monthly Landsat composites during the growing season have less than 50% cloud‐free pixels, while 21% of monthly growing season observations have no cloud‐free pixels at all (Figure S2). This is in comparison to MODIS, where just 3% of monthly composites have less than 50% cloud‐free pixels (Figure S1) and Sentinel‐2 with 14% of monthly growing season composites having less than 50% cloud‐free pixels (Figure S3). In the Rhӧn Biosphere Reserve, the longer revisit time of the Landsat sensors meant that the August NDVI distribution for 2018 is predominantly derived from a single image from the 6 August 2018 due to cloud cover obscuring the study forests in images captured later in the month. Given the lack of NDVI change observed in June and July 2018 by all the sensors tested here, it is likely that the canopy‐level expression of drought stress would have only just started to be observable in the Landsat image collected at the start of August 2018. As such, the cloud presence, combined with long revisit time, likely led to misrepresentation of the full monthly NDVI response to the 2018 drought event (Philipp *et al*. 2021). Consequently, satellite datasets with a shorter revisit time are essential to capture the canopy response to intense drought events and should be prioritized in the future to meet the requirement for early‐warning indicators of forest dieback and mortality (Camarero 2021).

The NDVI decline captured in the time‐series data was temporally lagged compared to the onset of the 2003 and 2018 drought events in the Rhӧn Biosphere Reserve by 1 and 2 months, respectively (Fig. 9). Early on in the growing season the forest canopy appears healthy in the remote sensing data until high temperatures and lack of moisture become limiting factors, causing a decline in canopy health and early senescence later in the summer, a temporal pattern confirmed across all three satellite datasets (Fig. 7). In the Rhӧn Biosphere Reserve, neither temperature nor precipitation alone elicit a consistent landscape‐scale response in NDVI of the European beech‐dominated forest canopy. However, the combination of intensely hot and dry weather, which is captured here by SPEI, produced conditions sufficient to result in a decline of canopy NDVI consistent with the early discolouration and senescence of Central European beech forests reported in the literature (Buras *et al*. 2020; Schuldt *et al*. 2020) (Fig. 9). The clearer association between landscape‐scale NDVI decline and hotter droughts has also been confirmed across German forests, with a stronger correlation coefficient found between NDVI response and the self‐calibrated Palmer Drought Severity Index and soil moisture estimates than with temperature (Philipp *et al*. 2021). Consequently, hotter drought events appear to have a more substantial impact on the canopy condition of European beech forests in the region than either high temperatures or low precipitation occurring in isolation.

The NDVI is used here because it is common to MODIS, Landsat and Sentinel‐2, allowing a comparison of the spatial and temporal properties of all three of these sensors at their highest spatial resolution. MODIS NDVI has been used for continental‐scale assessment of the 2018 drought (Buras *et al*. 2020), and NDVI‐based anomalies have shown greater correlation with drought indices in German forests than anomalies based on spectral indices using shortwave infrared spectral regions of the MODIS sensor (Philipp *et al*. 2021). However, there have been few side‐by‐side comparisons of Sentinel‐2, Landsat and MODIS for observing drought impacts on forest canopies. Here, we demonstrate that the same anomaly‐based method can be applied to Landsat or Sentinel‐2 data to identify spatial variability in canopy decline. While methods have been developed for Sentinel‐2 using phenology metrics to retrospectively classify areas of early senescene (Brun *et al*. 2020), our results confirm that anomaly‐based monitoring is robust across sensors and methods, such as the forest condition monitor for Germany (waldzustandsmonitor.de; Buras *et al*. 2020) or Bfast Monitor (Verbesselt *et al*. 2010), and could be adapted to use Sentinel‐2 in tandem with MODIS to identify hotspots of canopy decline in near‐real time. In doing so, the integration of multiple sources of remote sensing data into forest monitoring tools would facilitate more precise estimates of environmental change and improve our understanding of the drivers underlying the observed patterns (Mondal *et al*. 2020).

Future remote sensing observations of drought impact may be further improved by combining data from multiple sensors into virtual constellations to maximize temporal coverage, for example the Harmonized Landsat and Sentinel‐2 (HLS) products (Claverie *et al*. 2018; Moon *et al*. 2021). Advances in the harmonization of data products has led to improvement in the ability to characterize phenological cycles using the HLS dataset (Bolton *et al*. 2020; Moon *et al*. 2021). However, the HLS product contains fewer observations prior to 2018 than after, limiting its use for historical assessments and, as highlighted here, different greening trends between Landsat and Sentinel‐2 need to be carefully accounted for to ensure anomalies in vegetation indices are not misrepresented (Figs. 6 and 7; Bolton *et al*. 2020). Nonetheless, combining the time series of Landsat and Sentinel‐2 allows a high spatial resolution to be maintained while improving the temporal observation density. As the time series of Sentinel‐2 becomes longer, HLS, and potentially other sensor harmonizations, will provide exciting opportunities for improved phenological research and the needed early‐warning indicators of forest dieback and mortality.

Despite the ubiquity of NDVI, it has been shown to saturate with increasing biomass, leaf area index and leaf chlorophyll content (Huete *et al*. 1997; Morley *et al*. 2020). Given that the primary observable response of the forest canopy to intense drought is premature leaf discolouration and shedding, NDVI may limit our ability to detect early signals of canopy‐level discolouration because the saturation effect may mask subtle changes in canopy condition that occur before August (Vicca *et al*. 2016). An advantage of Sentinel‐2 is its inclusion of spectral bands in the red‐edge region of the electromagnetic spectrum (Smith *et al*. 2004). The red‐edge describes the spike in vegetation reflectivity between visible red and near infrared wavelengths (approximately 700–790 nm), and indices calculated using the red‐edge region have been found to be more sensitive to changes in chlorophyll content (Sims & Gamon 2002; Jung *et al*. 2006; Peng & Gitelson 2011; Schuster *et al*. 2012; Clevers & Gitelson 2013; Hawryło *et al*. 2018; Morley *et al*. 2020). Consequently, Sentinel‐2 provides additional potential to resolve subtle changes in chlorophyll content which may lead to an improved early assessment of premature leaf discolouration and senescence in European beech forests.

While it was not possible to characterize individual tree or stand‐level forest mortality in this study, ground‐based observations of mortality following early leaf senescence do exist in Central Europe (Brun *et al*. 2020; Schuldt *et al*. 2020), indicating that the areas with a high NDVI anomaly identified here could correspond to areas with increased tree mortality rates (Fig. 7). While the results presented here indicate a broad‐scale recovery of the European beech forest across the study area by 2020 (Fig. 4), tree mortality following drought events can be slow and spatially heterogeneous (Hlásny *et al*. 2015; Decuyper *et al*. 2020). Consequently, the green‐up of understorey vegetation and sub‐dominant trees that occurs as dominant canopy trees decline may substantially influence the canopy‐level assessment of persistent drought‐induced impacts or recovery. Since hotspots of tree mortality induced by intense drought can enable secondary infections of pests and pathogens to enter the forest, which can prove fatal for stressed forests (Dobbertin *et al*. 2001; Marini *et al*. 2017; Netherer *et al*. 2019), it is essential to link remote sensing observations with ground‐based surveys to better understand how NDVI anomalies are manifested in field‐based assessments of tree health and mortality (Dotzler *et al*. 2015). In doing so this would enable rapid quantification of drought‐induced canopy damage and help define early‐warning indicators for forest mortality (Decuyper *et al*. 2020).

## CONCLUSION

European beech forests are at risk from intense drought events. The NDVI response from canopy data in an area predicted to be suitable for European beech under future climate scenarios has been severely impacted by the intense drought events of 2003 and 2018. When using MODIS time series NDVI data to examine beech forest canopy response to intense drought, we find that the temporal development of these extreme droughts can be observed to start in July, become most clear in August and persist through September in each case. Despite this consistent pattern, the canopy response lagged behind the onset of the drought by up to 2 months. The choice of sensor used to observe drought impacts has an important influence on the impacts that can be observed and, to improve monitoring of drought‐induced canopy decline, multiple sensors should be used in tandem to understand how intense drought events impact spatial and temporal patterns of canopy decline. While the majority of drought studies to date utiliae MODIS’s consistent repeat measurements to identify temporal patterns of canopy declines, this sensor lacks the pixel size for identifying spatial variation in canopy decline. In contrast, Sentinel‐2 provides a high‐resolution understanding of spatial variation in drought impacts, despite lacking a long historical legacy. Consequently, incorporating multiple data sources is essential to gain both a temporal and spatial understanding of drought‐induced impacts on forest canopies. By combining data sources, the methods presented in this paper will enable the improved monitoring of tree canopy impacts in response to intense drought events over time and space. This will facilitate understanding of drivers of spatial and temporal variation that are critical to better predict how these forests will respond to future climate projections.

## Supporting information


**Figure S1**. Monthy median time‐series NDVI data derived from MODIS 8‐day composites (MOD09Q1 v.6) showing the proportion of cloud free pixels within the study area in each month (A), the median NDVI response for the broadleaf forests within the Rhӧn Biosphere Reserve core protected areas with 25^th^ and 75^th^ percentiles shaded (B) and the monthly NDVI deviation from the 20 year monthly average with 25^th^ and 75^th^ percentiles shaded (C). The intense drought years of 2003 and 2018 are shaded in grey.
**Figure S2**. Monthy median time‐series NDVI data derived from Landsat series showing the proportion of cloud free pixels within the study area in each month (A), the median NDVI response for the broadleaf forests within the Rhӧn Biosphere Reserve core protected areas with 25^th^ and 75^th^ percentiles shaded (B) , the median NDVI response after detrending with least‐squares regression with 25^th^ and 75^th^ percentiles shaded (C) and the monthly NDVI deviation from the 20 year monthly average with 25^th^ and 75^th^ percentiles shaded (D). The intense drought years of 2003 and 2018 are shaded in grey.
**Figure S3**. Monthy median time‐series NDVI data derived from Sentinel‐2 MSI showing the proportion of cloud free pixels within the study area in each month (A), the median NDVI response for the broadleaf forests within the Rhӧn Biosphere Reserve core protected areas with 25th and 75th percentiles shaded (B) and the monthly NDVI deviation from the four year monthly average with 25^th^ and 75^th^ percentiles shaded (C). The intense drought year of 2018 is shaded in grey.
