# Supplementary material for: Satellite data track spatial and temporal declines in European beech forest canopy characteristics associated with intense drought events in the Rhön Biosphere Reserve, central Germany
Source: Plant Biol (Stuttg). 2022 Jan 27;24(7):1120–31. doi: 10.1111/plb.13391 (PMC10078791; doi:10.1111/plb.13391)
Supplement: Supplementary file 1 — Figure S1. Monthy median time‐series NDVI data derived from MODIS 8‐day composites (MOD09Q1 v.6) showing the proportion of cloud free pixels within the study area in each month (A), the median NDVI response for the broadleaf forests within the Rhӧn Biosphere Reserve core protected areas with 25th and 75th percentiles shaded (B) and the monthly NDVI deviation from the 20 year monthly average with 25th and 75th percentiles shaded (C). The intense drought years of 2003 and 2018 are shaded in grey. Figure S2. Monthy median time‐series NDVI data derived from Landsat series showing the proportion of cloud free pixels within the study area in each month (A), the median NDVI response for the broadleaf forests within the Rhӧn Biosphere Reserve core protected areas with 25th and 75th percentiles shaded (B) , the median NDVI response after detrending with least‐squares regression with 25th and 75th percentiles shaded (C) and the monthly NDVI deviation from the 20 year monthly average with 25th and 75th percentiles shaded (D). The intense drought years of 2003 and 2018 are shaded in grey. Figure S3. Monthy median time‐series NDVI data derived from Sentinel‐2 MSI showing the proportion of cloud free pixels within the study area in each month (A), the median NDVI response for the broadleaf forests within the Rhӧn Biosphere Reserve core protected areas with 25th and 75th percentiles shaded (B) and the monthly NDVI deviation from the four year monthly average with 25th and 75th percentiles shaded (C). The intense drought year of 2018 is shaded in grey. [file PLB-24-1120-s001.docx]

# Supplimentary Information: Satellite data tracks spatial and temporal declines in European beech forest canopy characteristics associated with intense drought events in the Rhön Biosphere Reserve, Central Germany

Emily West^a^, Peter J. Morley^a,b^ *, Alistair S. Jump^b^ , Daniel N.M. Donoghue^a^


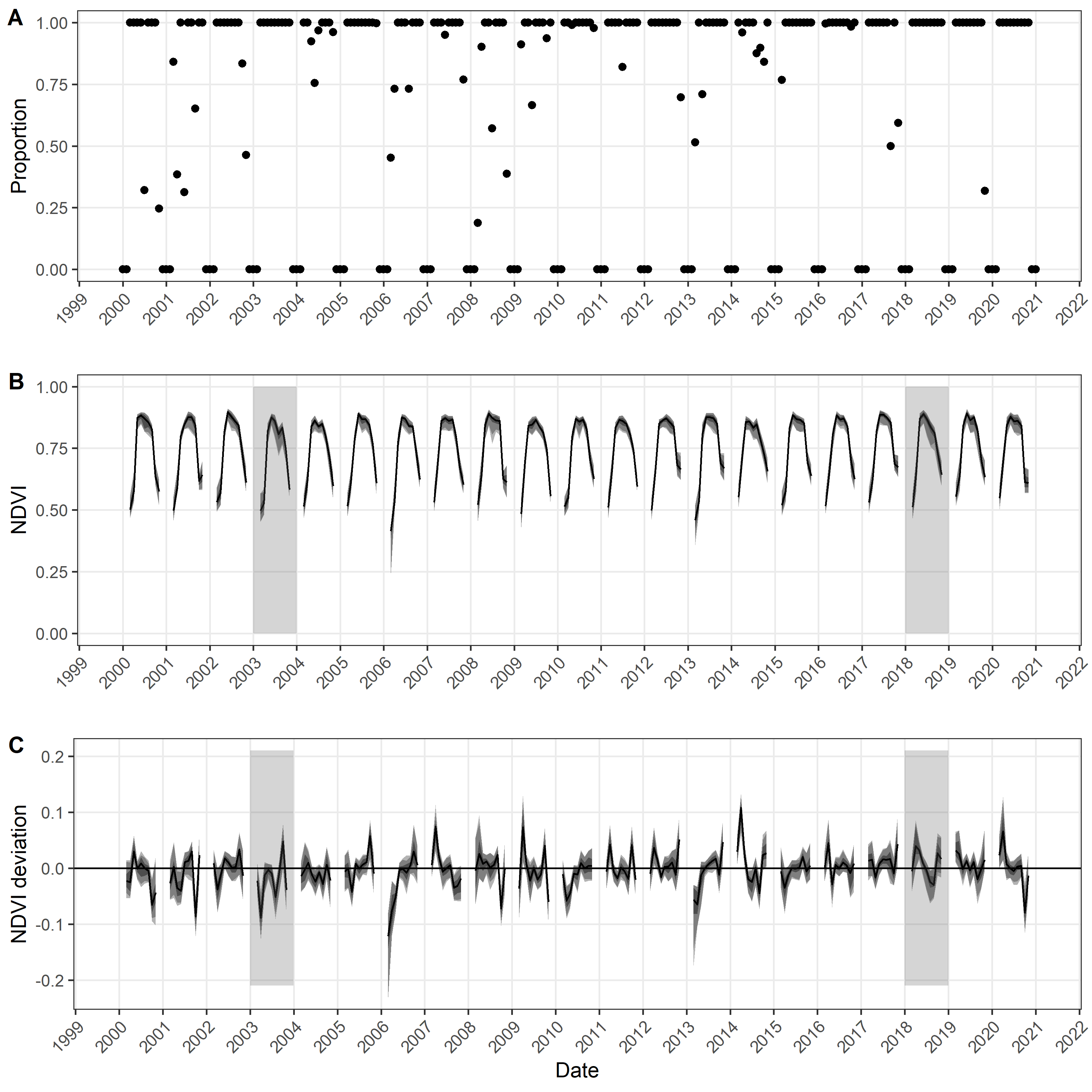


Figure S1: Monthy median time-series NDVI data derived from MODIS 8-day composites (MOD09Q1 v.6) showing the proportion of cloud free pixels within the study area in each month (A), the median NDVI response for the broadleaf forests within the Rhӧn Biosphere Reserve core protected areas with 25^th^ and 75^th^ percentiles shaded (B) and the monthly NDVI deviation from the 20 year monthly average with 25^th^ and 75^th^ percentiles shaded (C). The intense drought years of 2003 and 2018 are shaded in grey.


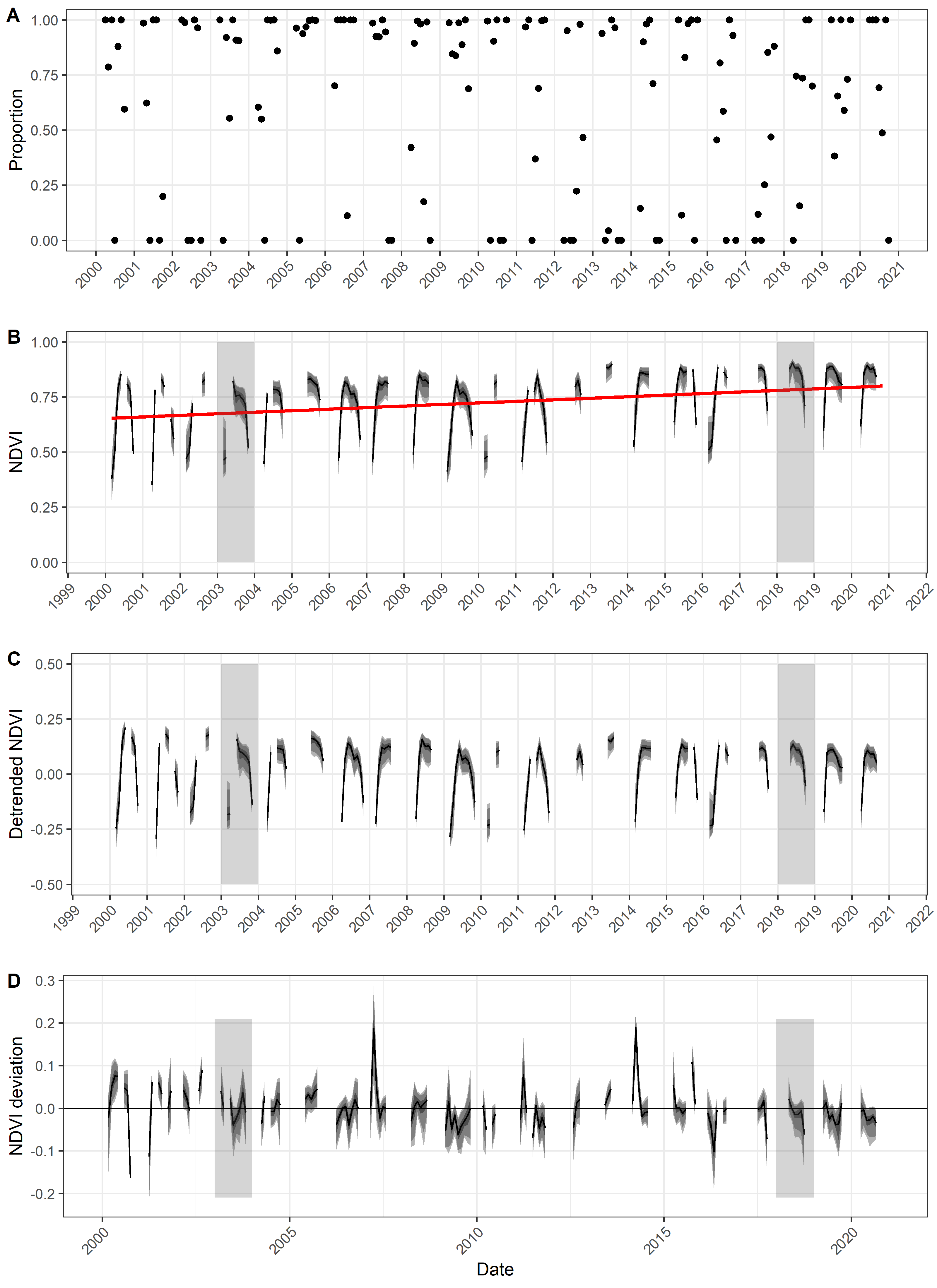


Figure S2: Monthy median time-series NDVI data derived from Landsat series showing the proportion of cloud free pixels within the study area in each month (A), the median NDVI response for the broadleaf forests within the Rhӧn Biosphere Reserve core protected areas with 25^th^ and 75^th^ percentiles shaded (B) , the median NDVI response after detrending with least-squares regression with 25^th^ and 75^th^ percentiles shaded (C) and the monthly NDVI deviation from the 20 year monthly average with 25^th^ and 75^th^ percentiles shaded (D). The intense drought years of 2003 and 2018 are shaded in grey.


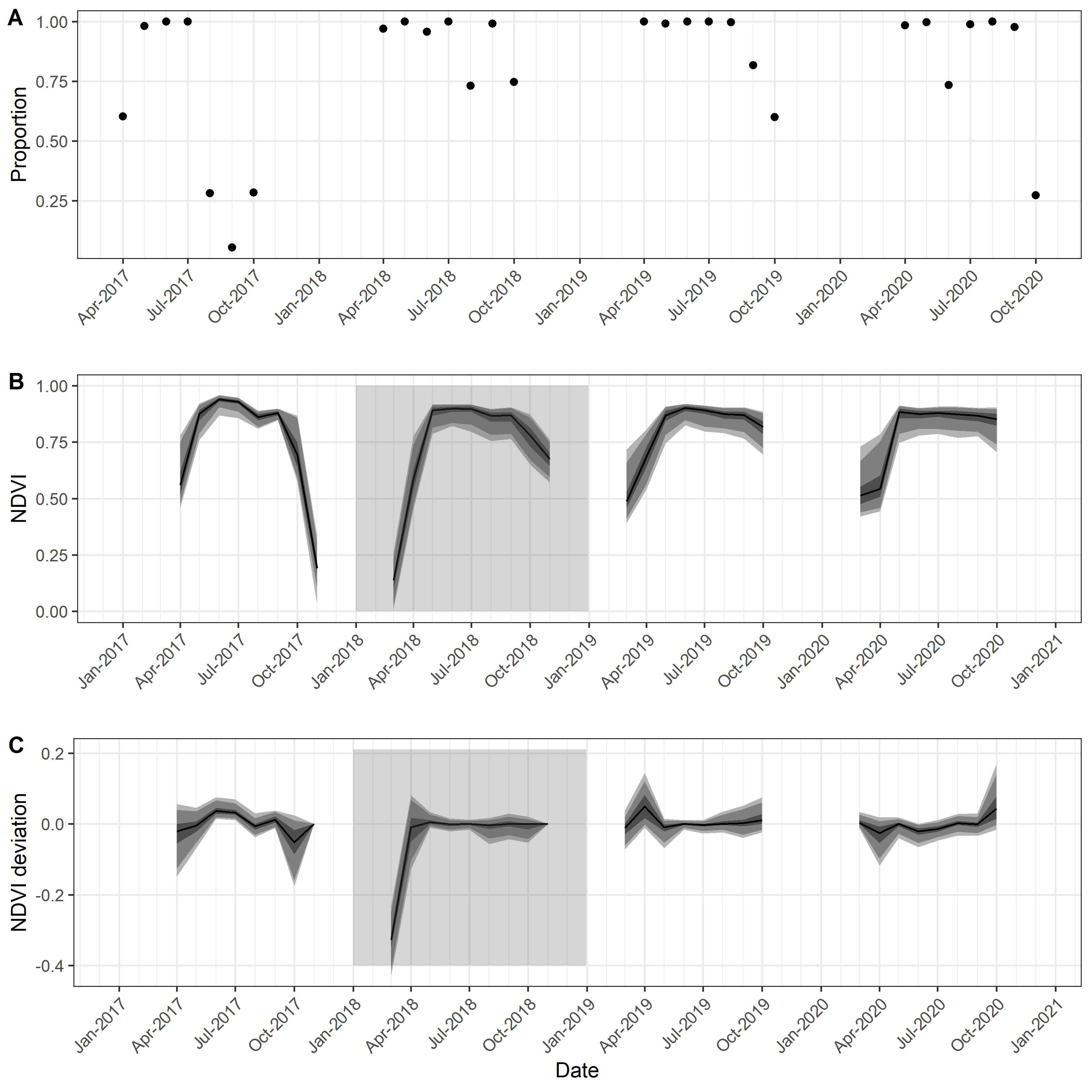


Figure S1: Monthy median time-series NDVI data derived from Sentinel-2 MSI showing the proportion of cloud free pixels within the study area in each month (A), the median NDVI response for the broadleaf forests within the Rhӧn Biosphere Reserve core protected areas with 25^th^ and 75^th^ percentiles shaded (B) and the monthly NDVI deviation from the four year monthly average with 25^th^ and 75^th^ percentiles shaded (C). The intense drought year of 2018 is shaded in grey.
